# Resveratrol Inhibits Insulin-Induced Vascular Smooth Muscle Cell Proliferation and Migration by Activating SIRT1

**DOI:** 10.1155/2022/8537881

**Published:** 2022-11-28

**Authors:** Yijie Wang, Lifu Lei, Qian Su, Si Qin, Jian Zhong, Yinxing Ni, Jian Yang

**Affiliations:** ^1^Department of Endocrinology, The Third Affiliated Hospital of Chongqing Medical University, Chongqing 401120, China; ^2^Research Center for Metabolic and Cardiovascular Disease, The Third Affiliated Hospital of Chongqing Medical University, Chongqing 401120, China; ^3^Department of Clinical Nutrition, The Third Affiliated Hospital of Chongqing Medical University, Chongqing 401120, China

## Abstract

Abnormal proliferation and migration of vascular smooth muscle cells (VSMCs) are essential for the development of hypertension. Insulin has been identified to promote VSMC proliferation and migration; resveratrol has been shown to have protective effects against cardiovascular diseases. This study aimed to investigate the effect of resveratrol on insulin-induced VSMC proliferation and migration and its potential mechanism. VSMC proliferation was measured by Cell Counting Kit-8 (CCK-8), cell counting method, and 5-ethynyl-2′-deoxyuridine (EdU) incorporation assay. Cell migration was detected by wound healing assay and transwell method. Expression of silent information regulator of transcription 1 (SIRT1) and phosphorylation levels of signaling molecules, such as phosphatidylinositol 3-kinase (PI3K) and protein kinase B (Akt), in VSMCs were detected by Western blotting. Resveratrol (25-150 *μ*M) was found to inhibit insulin-induced VSMC proliferation. Pretreatment with 100 *μ*M resveratrol reduced insulin (100 nM)-mediated VSMC migration. LY294002, an inhibitor of PI3K, inhibited the stimulatory effect of insulin (100 nM) on the proliferation of VSMCs. Treatment with resveratrol also decreased insulin-induced stimulatory effect on PI3K and Akt phosphorylation levels. Moreover, resveratrol treatment increased SIRT1 protein expression in VSMCs. A SIRT1 inhibitor, EX527, reversed the inhibitory effect of resveratrol on insulin-induced VSMC proliferation and migration and activation of PI3K and Akt phosphorylation levels. In conclusion, our study revealed that treatment with resveratrol inhibited insulin-mediated VSMC proliferation and migration, possibly by activating SIRT1 and downregulating the PI3K/AKT pathway.

## 1. Introduction

Hypertension, a major risk factor for morbidity and mortality in patients with cardiovascular diseases, is considered a global public health concern with a huge economic burden [1–3]. Many factors, including increased oxidative stress, damage of autonomic nervous system, and activation of the renin-angiotensin-aldosterone system, have been reported to result in the development of hypertension [4–6]. Among them, abnormal proliferation and migration of vascular smooth muscle cells (VSMCs) play vital roles in the pathogenesis of hypertension [7].

Insulin resistance is associated with an increased risk of hypertension [8]. Patients with insulin resistance and hypertension have a four-fold higher cardiovascular disease risk than those with normal blood glucose and blood pressure. Hyperinsulinemia, a notable clinical manifestation in patients with insulin resistance, is an independent risk factor and an indicator of poor prognosis of hypertension and other cardiovascular diseases in patients with insulin resistance [9]. Studies have shown that insulin promotes the proliferation and migration of VSMCs, leading to vascular remodeling and neointima formation, subsequently causing hypertension and other cardiovascular diseases [10, 11]. Therefore, development of an effective strategy to avoid hyperinsulinemia-induced cardiovascular complications would be essential.

To improve medication adherence and risk factor prevention in patients with hypertension, strengthening the health belief model with continuous and ongoing education about hypertension is essential [12]. Lifestyle factors, including diet, physical activity, alcohol consumption, and cigarette smoking, are associated with hypertension. Nonpharmacological methods, such as lifestyle modifications, would aid the prevention and therapy of hypertension [13]. Studies have shown that nutrients in food, including fiber, *n*-3 fatty acids, and some phytochemicals, exert beneficial physiological effects in hypertensive subjects and animal models [14–16]. Among the nutrients, phytochemicals have attracted much attention recently. Resveratrol, a natural polyphenolic compound, is widely found in food, such as grapes and red wine [17]. It exerts antioxidant, anti-inflammatory, and cardioprotective effects by activating sirtuins [18]. For example, resveratrol inhibits the high glucose-induced migration of VSMCs, ameliorates endothelial injury of the thoracic aorta in diabetic mice, reduces renal inflammation, and attenuates the progression of hypertension in spontaneously hypertensive rats (SHRs) [19–21]. However, the effect of resveratrol on insulin-induced vascular injury remains unclear. In the present study, we aimed to investigate the effect of resveratrol on insulin-induced VSMC proliferation and migration and determine the underlying mechanisms.

## 2. Materials and Methods

### 2.1. Cell Culture

The A10 cell line, a rat thoracic aorta smooth muscle cell line, was obtained from the American Type Culture Collection (ATCC, Manassas, VA, USA) and cultured in Dulbecco's modified Eagle's medium (DMEM, Gibco, Waltham, MA, USA) supplemented with 10% fetal bovine serum (FBS, Gibco, Waltham, MA, USA) and 1% penicillin and streptomycin. Cells were maintained at 37°C in a 5% CO_2_ incubator, and when grown to 80-90% confluence, they were passaged at a ratio of 1 : 3. The VSMCs were incubated in serum-free DMEM for 2 h before being exposed to different insulin concentrations or resveratrol for the indicated times, depending on the experiment.

### 2.2. Cell Counting Kit-8 (CCK-8) Assay

Cells were subcultured in 96-well plates (1.0 × 10^4^ cells per well) and treated with various concentrations of insulin or/and resveratrol for 24 h. To determine cell proliferation, 10 *μ*L of Cell Counting Kit-8 solution (CCK-8, Bimake, Houston, TX, USA) was added to each well of the plate, which was then incubated at 37°C for an additional 2 h. Absorbance was read at 450 nm using a microplate reader (SYNERGY H1, BioTek, Winooski, VT, USA).

### 2.3. Cell Counting Assay

VSMCs were seeded into 12-well plastic culture plates at a density of 3.0 × 10^4^ cells per well in DMEM containing 10% FBS at 37°C. When the cells reached 70% confluence, the medium was changed to a serum-free medium and the cells were further incubated for 2 h. VSMCs were pretreated with various concentrations of resveratrol for 30 min and then stimulated with 100 nM insulin for another 24 h. After incubation, the cells were trypsinized with trypsin-EDTA and counted using a Countstar automated cell counter (Alit Life Science Co. Limited, Shanghai, China). The count was repeated five times for each sample.

### 2.4. 5-Ethynyl-2′-deoxyuridine (EdU) Incorporation Assay

After drug treatment, the cells were incubated in 96-well plates (1.0 × 10^4^ cells per well) with 50 *μ*M EdU medium for 2 h. According to the EdU incorporation assay (RiboBio, Guangzhou, China) protocol, 4% paraformaldehyde was added for 30 min to treat the cells. To each well, 100 *μ*L of 1× Apollo® staining reaction solution was added, and the cells were incubated with 100 *μ*L of 1× Hearst 33342 reaction solution. The cells were photographed and counted in any of the five areas selected under the fluorescence microscope. The EdU positivity rate was calculated as (EdU incorporated cells/Hoechst-stained cells) × 100%.

### 2.5. Wound Healing Assay

Cells were inoculated in 6-well plates at a density of 1.0 × 10^4^ cells in 200 *μ*L per well. The monolayer cells were vertically scribed with a 200-*μ*L sterile cell scraper when they reached full-density saturation. After washing, the medium was replaced with serum-free medium containing insulin, resveratrol (Sigma-Aldrich, R5010), or EX527 (MedChemExpress, HY-15452), according to the experimental scheme, and the cells were further incubated for 24 h. The cells were observed and photographed at 0 h and 24 h, and the scratch area was measured using image analysis software to calculate the scratch healing rate. Scratch healing rate = (0 h scratch area − 24 h scratch area)/0 h scratch area × 100%.

### 2.6. Transwell Migration Assay

Transwell migration assays were performed in 24-well plates (Corning Incorporated, Corning, NY, USA), using 8.0-*μ*m Transwell Permeable Supports (BD Falcon, Franklin Lakes, NJ, USA). Cells in the logarithmic growth phase were added to the upper chamber of the transwell at 1.0 × 10^4^ cells per well in 200 *μ*L of supplemented DMEM. After incubation, the medium in the upper chamber was replaced with serum-free medium containing resveratrol or EX527, and cell migration was induced by 100 *μ*M insulin in the lower chamber. The chamber was then incubated at 37°C for 24 h, fixed in paraformaldehyde for 1 h, air-dried, and stained with crystal violet for 30 min. Five randomly selected fields of view were photographed using a microscope. Image J software was used to calculate the number of membrane-penetrating cells, and ratio of the number of membrane-penetrating cells in the experimental group to that in the control group represented the change in cell migration ability.

### 2.7. Western Blotting

Total cellular proteins were extracted using radioimmunoprecipitation assay (RIPA) lysis buffer containing protease and phosphatase inhibitor cocktails (Bimake, Houston, TX, USA). Thereafter, the supernatant was collected for quantifying the total cellular protein using a bicinchoninic acid (BCA) protein content assay kit (BCA kit, Beyotime Biotechnology, Shanghai, China). Next, the protein was denatured and inactivated in a metal bath at 100°C for 5 min. Equal amounts of protein (30 *μ*g) were loaded onto sodium dodecyl sulfate polyacrylamide gel for electrophoresis and then transferred to PVDF membranes using a transfer system. The blots were blocked with 5% skimmed milk for 2 h and then incubated overnight at 4°C with the corresponding primary antibody. After washing thrice with TBST, the blots were incubated with the corresponding secondary horseradish peroxidase (HRP)-conjugated antibody at room temperature for 1 h. Finally, the ECL luminescent solution (WBKLS0500, Millipore, Burlington, MA, USA) was used to visualize and photograph the immunoreactive proteins. Density of the bands in the images was quantified using Image J software.

### 2.8. Statistical Analysis

All statistical data are presented as the mean ± standard deviation (SD). Student's *t*-test was used to determine statistical significance of the differences between two groups. Statistical significance was set at *P* < 0.05.

## 3. Results

### 3.1. Inhibition of Insulin-Induced VSMC Proliferation by Resveratrol

**Figure 1:**
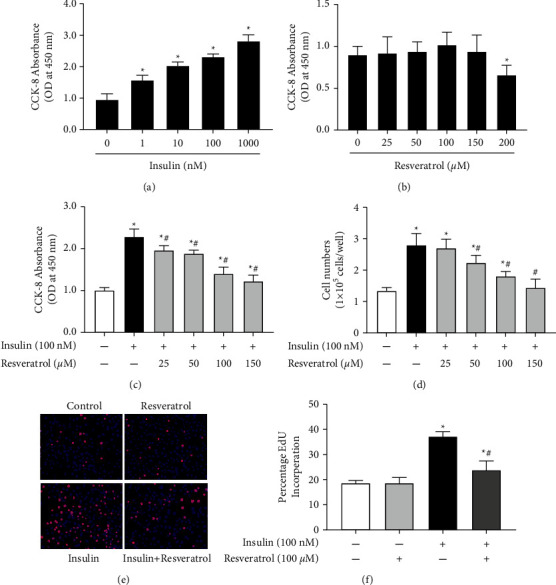
Resveratrol inhibits insulin-induced VSMC proliferation. (a) Effect of insulin on VSMC proliferation. After incubating VSMCs with various insulin concentrations for 24 h, CCK-8 assay was performed to assess the OD values at 450  nm for each treatment group. ^*∗*^*P* < 0.05 vs. control, *n* = 5/group. (b) Effect of resveratrol on VSMC proliferation. A10 cells were treated with different concentrations of resveratrol (25-200 *µ*M) for 24 h. Cell proliferation was quantified using the CCK-8 method and compared with that of the control group. ^*∗*^*P* < 0.05 vs. control, *n* = 5/group. (c) and (d) effect of resveratrol on insulin-induced proliferation of VSMCs. Cell proliferation was detected using the CCK-8 assay (c) or direct cell counting (d) after incubation with insulin (100 nM) in the absence or presence of resveratrol (25-200 *μ*M). ^*∗*^*P* < 0.05 vs. control; ^#^*P* < 0.05 vs. insulin group, *n* = 6/group. (e) and (f) EdU incorporation assay was performed to detect the effect of resveratrol (100 *μ*M) on VSMC proliferation after pretreatment with insulin (100 nM). (e) DNA synthesis in VSMCs was tested by EdU incorporation. (f) The ratio of EdU-positive cells to total cells. ^*∗*^*P* < 0.05 vs. control; ^#^*P* < 0.05 vs. insulin group, *n* = 9/group.

To quantify the effect of insulin on the proliferation of VSMCs, A10 cells were treated with different concentrations of insulin for 24 h. Results showed that insulin promoted VSMC proliferation in a concentration-dependent manner (Figure 1(a)). Treatment with resveratrol at concentrations in the range of 25-150 *μ*M had no effect on VSMC proliferation (Figure 1(b)). However, resveratrol attenuated insulin-induced VSMC proliferation in a dose-dependent manner (Figure 1(c)). The inhibitory effect was verified by the cell counting method (Figure 1(d)) and EdU incorporation assay (Figures 1(e) and 1(f)). Therefore, resveratrol was found to inhibit insulin-induced VSMC proliferation.

### 3.2. Inhibition of Insulin-Induced VSMC Migration by Resveratrol

**Figure 2:**
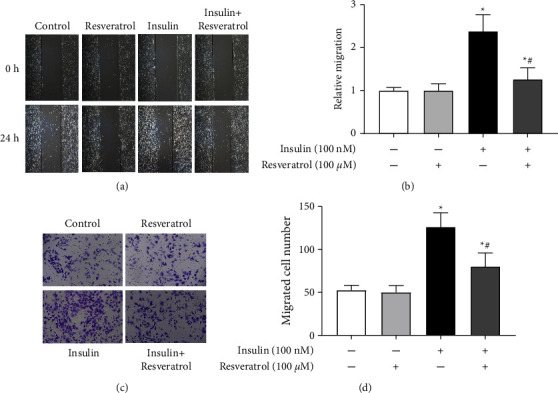
Effects of resveratrol on insulin-induced VSMC migration. (a) and (b) Effects of resveratrol on insulin-induced VSMC migration were measured via wound healing assay. Measurements were made 24 h after treatment with vehicle, resveratrol (100 *μ*M), or/and insulin (100 nM). Images were taken at regular intervals (a) and the relative migration rate was calculated (b). ^*∗*^*P* < 0.05 vs. control; ^#^*P* < 0.05 vs. insulin group, *n* = 6/group. (c) and (d) Effects of resveratrol on insulin-induced VSMC migration were measured by transwell assays. (c) Representative pictures of cells that migrated through the filter and were stained with crystal violet. (d) Bar graph showing the number of migrated VSMCs. ^*∗*^*P* < 0.05 vs. control; ^#^*P* < 0.05 vs. insulin group, *n* = 6/group.

The effect of resveratrol on insulin-induced VSMC migration was analyzed next. Results of the wound healing assay showed the migration rate to be higher in the insulin-stimulated group than in the control group; however, treatment with resveratrol inhibited insulin-induced cell migration remarkably (Figures 2(a) and 2(b)). Transwell assay also suggested that VSMC migration was increased after treatment with insulin and was inhibited by resveratrol (Figures 2(c) and 2(d)). Overall, resveratrol treatment was found to reduce insulin-mediated VSMC migration.

### 3.3. Role of PI3K in the Effect of Insulin on VSMC Proliferation

**Figure 3:**
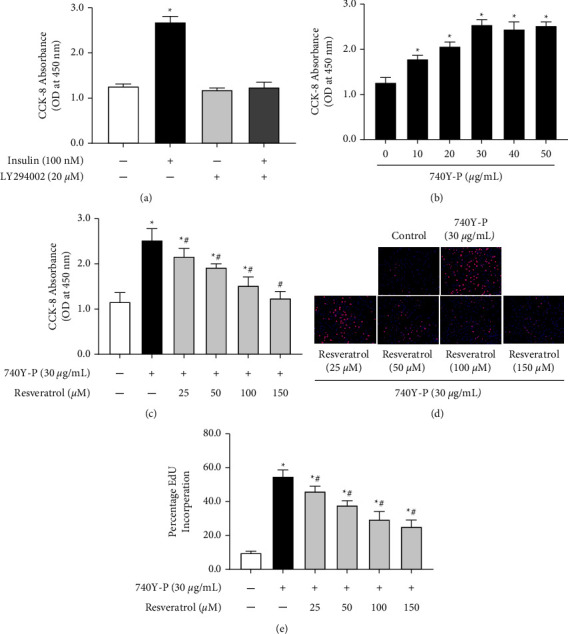
Role of PI3K in the effect of insulin-induced VSMC proliferation. (a) Effect of LY294002 on insulin-induced VSMC proliferation. Cells were treated with insulin (100 nM) in the presence of PI3K inhibitors (LY294002, 20 *μ*M) for 24 h. CCK-8 assay evaluated the OD values at 450 nm for each treatment group. ^*∗*^*P* < 0.05 vs. others, *n* = 9/group. (b) Effect of 740Y-P on VSMC proliferation. A10 cells were incubated with various doses of PI3K activator, 740Y-P (10-50 *μ*g/mL) for 24 h. CCK-8 assay measured the proliferation of VSMCs using OD values at 450 nm. ^*∗*^*P* < 0.05 vs. control, *n* = 9/group. (c) VSMCs were treated with 740Y-P (30 *μ*g/mL) with or without resveratrol (100 *μ*M) for 24 h. Proliferation of VSMCs was analyzed by CCK-8 assay. Results are expressed as optical density. ^*∗*^*P* < 0.05 vs. control; #*P* < 0.05 vs. 740Y–P group, *n* = 9/group. (d) and (e) VSMCs were treated with 740Y-P (30 *μ*g/mL) with or without resveratrol (100 *μ*M) for 24 h. Proliferation of VSMCs was analyzed by the EdU incorporation assay. (d) DNA synthesis in VSMCs was determined using EdU incorporation assay. (e) The ratio of EdU-positive cells to total cells. ^*∗*^*P* < 0.05 vs. control; ^#^*P* < 0.05 vs. insulin group, *n* = 6/group.

To investigate the role of PI3K in insulin-induced VSMC proliferation, LY294002, an inhibitor of PI3K, was used. LY294002 had no substantial effect on the proliferation of VSMCs, although it inhibited the stimulatory effect of insulin on VSMC proliferation (Figure 3(a)). Moreover, treatment with 740Y-P, a PI3K activator, elevated VSMC proliferation in a dose-dependent manner (Figure 3(b)). To determine the effect of resveratrol on VSMC proliferation stimulated by 740Y-P, we measured cell proliferation after treatment with resveratrol combined with 740Y-P. CCK-8 assay results showed that resveratrol reversed the 740Y-P-induced VSMC proliferation (Figure 3(c)), which was then confirmed by the EdU incorporation assay (Figures 3(d) and 3(e)).

### 3.4. Inhibition of Insulin-Induced PI3K/Akt Activation in VSMC Proliferation due to Resveratrol

**Figure 4:**
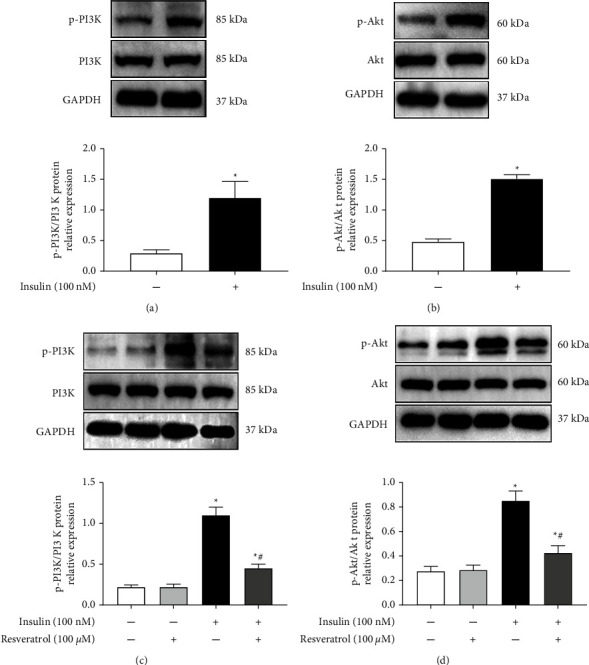
Role of resveratrol in the effect of insulin-induced PI3K/Akt activation (a) and (b) effect of insulin on PI3K signaling pathway in VSMCs. A10 cells were incubated with insulin (100 nM) for 30 min, and Western blotting was performed to analyze PI3K and Akt phosphorylation levels. Relative phosphorylation levels of PI3K and Akt were quantified. ^*∗*^*P* < 0.05 vs. control, *n* = 5/group. (c) and (d) Effect of resveratrol on insulin-induced PI3K/Akt activation in VSMCs. Western blotting identified the protein expression levels of p-PI3K and p-Akt, whether or not resveratrol 100 *μ*M was pretreated. Bar graph showing the relative protein expression of phosphorylated and total PI3K and Akt. ^*∗*^*P* < 0.05 vs. control; #*P* < 0.05 vs. insulin group, *n* = 5/group.

Role of PI3K/Akt pathway in the inhibitory effect of resveratrol on insulin-induced VSMC proliferation was examined. Results showed that treatment with insulin increased the PI3K and Akt phosphorylation levels remarkably in VSMCs (Figures 4(a) and 4(b)). However, resveratrol reduced the insulin-mediated stimulatory effect on the phosphorylation levels of PI3K and Akt (Figures 4(c) and 4(d)). Taken together, the PI3K/Akt pathway was involved in the inhibitory effect of resveratrol on insulin-induced VSMC proliferation.

### 3.5. Role of SIRT1 in the Inhibitory Effect of Resveratrol on Insulin-Induced VSMC Proliferation

**Figure 5:**
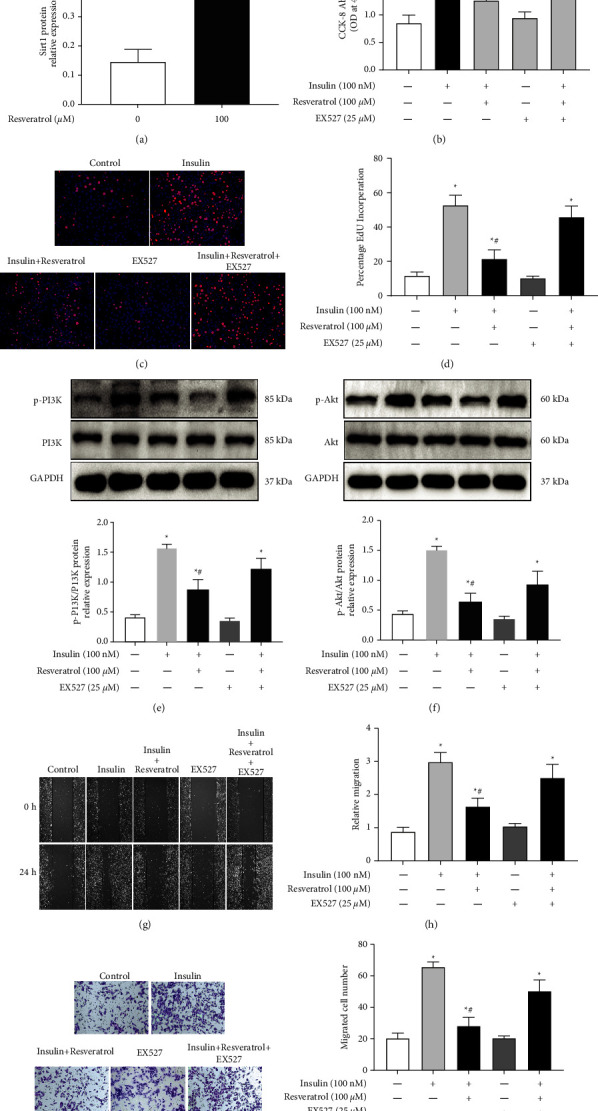
Role of SIRT1 in the inhibitory effect of resveratrol on insulin-induced VSMC proliferation. (a) Effect of resveratrol on SIRT1 expression. VSMCs were incubated with 100 *μ*M resveratrol for 24 h. SIRT1 protein levels were determined by Western blotting. ^*∗*^*P* < 0.05 vs. control, *n* = 5/group. (b) Cell proliferation after resveratrol treatment was quantified by the CCK-8 assay in insulin-treated cells that had been treated with or without the SIRT1 inhibitor EX-527. Results are expressed as optical density. ^*∗*^*P* < 0.05 vs. control; ^#^*P* < 0.05 vs. insulin group, *n* = 9/group. (c) and (d) EdU assays were performed to assess the effect of SIRT1 inhibitor EX527 on VSMC proliferation. (c) DNA synthesis in VSMCs was determined by EdU incorporation assay. (d) The ratio of EdU-positive cells to total cells. ^*∗*^*P* < 0.05 vs. control; ^#^*P* < 0.05 vs. insulin group, *n* = 6/group. (e) and (f) A10 cells were treated with the blank, insulin (100 nM), resveratrol (100 *μ*M), EX527 (25 *μ*M), or both insulin (100 nM) and resveratrol (100 *μ*M). Western blotting was performed to analyze PI3K and Akt phosphorylation levels. (e) Representative images and relative protein expression of p-PI3K. (f) Representative images and the relative protein expression of p-Akt. ^*∗*^*P* < 0.05 vs. control; ^#^*P* < 0.05 vs. insulin group, *n* = 5/group. (g) and (h) Wound healing assay was performed to measure the regulation of SIRT1 inhibitor EX527 in the effect of resveratrol on insulin-induced VSMC migration. (g) Images were taken at regular intervals. (h) Relative migration rate was calculated. ^*∗*^*P* < 0.05 vs. control; ^#^*P* < 0.05 vs. insulin group, *n* = 6/group. (i) and (j) Transwell assay was performed to measure the regulation of SIRT1 inhibitor EX527 in the effect of resveratrol on insulin-induced VSMC migration. (i) Representative pictures of cells that migrated through the filter and were stained with crystal violet. (j) Bar graph showing the number of migrated VSMCs. ^*∗*^*P* < 0.05 vs. control; ^#^*P* < 0.05 vs. insulin group, *n* = 6/group.

Role of SIRT1 in the inhibitory effect of resveratrol on insulin-induced VSMC proliferation was investigated next. Treatment with resveratrol enhanced the SIRT1 protein expression in VSMCs (Figure 5(a)). The SIRT1 inhibitor EX527 had no effect on VSMC proliferation but reversed the inhibitory effect of resveratrol on insulin-induced VSMC proliferation (Figure 5(b)), as verified by the EdU incorporation assay (Figures 5(c) and 5(d)). Further studies found that treatment with EX527 alone had no effect on the phosphorylation of PI3K and Akt, but reversed the inhibitory effect of resveratrol on insulin-induced phosphorylation of the two signaling molecules (Figures 5(e) and 5(f)). Additionally, EX527 blocked the inhibitory effect of resveratrol on insulin-induced migration of VSMCs (Figures 5(g)–5(j)).

## 4. Discussion

There is consensus among national and international experts that hypertension is the leading cause of death in patients with cardiovascular diseases [22]. Elevated blood pressure and vascular reactivity are associated with insulin resistance, which increases the risk of cardiovascular death by two to three times [23]. Accumulating evidence has demonstrated that long-term hyperinsulinemia is directly involved in the hypertension process, which ultimately leads, directly or indirectly, to cardiovascular events [24]. Circulating insulin level is elevated in subjects with hypertension [25]. Studies have shown that insulin accelerates VSMC proliferation and migration, leading to the development of hypertension [26, 27]. Inhibition of insulin-mediated VSMC proliferation may play an important role in the regulation of blood pressure [28]. In our present study, we confirmed that treatment with insulin induces the proliferation and migration of A10 cells, a rat thoracic aorta smooth muscle cell line [29]. Other studies have also shown similar effects in VSMCs from other species, such as humans and mice [30, 31].

Phytochemicals have been widely used in pharmaceutical and dietary treatments in recent years [32]. Resveratrol, a nonflavonoid polyphenolic compound found in grapes, Thuja, peanuts, and many other plants, mediating anti-inflammatory effects and inhibiting the function of cyclooxygenase and hydroperoxidase, is widely used in research on cardiovascular protection [33]. Epidemiological research has proven that resveratrol can prevent various diseases, including atherosclerosis and other cardiovascular diseases [34]. Resveratrol exerts some physiological functions in VSMCs, including inhibition of high glucose-induced migration mediated by focal adhesion kinase and attenuation of homocysteine-mediated cell proliferation [20, 35]. However, the effect of resveratrol on insulin-induced VSMC proliferation and migration remains unclear. Therefore, we investigated whether resveratrol could reduce the proliferation and migration of VSMCs. Our results showed that resveratrol is less cytotoxic to VSMCs when the concentration is below 200 *μ*M and significantly inhibits insulin-induced proliferation and migration at a concentration of 100 *μ*M. Resveratrol may exert broad inhibition on the effects induced by many hormones. For example, administration of resveratrol suppresses VSMC senescence [36], hypertrophy [37], and proliferation [38] induced by angiotensin II (Ang II). In addition, studies have shown that treatment with insulin not only promotes VSMC proliferation and migration but also induces VSMC phenotype conversion [31] and acid production [39], inhibiting gap junction intercellular communication in VSMCs [40]. Whether resveratrol can attenuate other effects mediated by insulin in VSMCs needs to be explored in future. While only A10 cell line was used in our present study, it would be better to use more cell lines to confirm the results of the current study. Moreover, whether resveratrol exerts similar effect in VSMCs from other species, such as humans and mice, would need to be explored further.

Insulin is a pleiotropic hormone that regulates many key physiological responses, including cell proliferation and differentiation [41]. The phosphatidylinositol PI3K/Akt pathway is the classical signaling pathway that mediates insulin action and is responsible for most of the metabolic activities of insulin [42, 43]. In our present study, we found that insulin induces proliferation and migration of VSMCs through PI3K/Akt signaling activation, which is consistent with previous reports [44]. Further investigation showed that the presence of resveratrol inhibits insulin-induced VSMC proliferation and migration, which are accompanied by reduced phosphorylation levels of PI3K and Akt. The findings indicated that the PI3K/Akt pathway may, at least in part, be involved in the inhibitory effect of resveratrol on the insulin-mediated proliferation and migration of VSMCs. However, besides the PI3K/Akt pathway, insulin also exerts its functions through other signaling pathways, including ERK1/2 MAPK signaling [44] and cytosolic phospholipase 2 (cPLA2) [45]. Thus, resveratrol could possibly inhibit other insulin-mediated signaling pathways as well, in VSMCs, which might have influenced the results of the present study. This would, however, need further investigation.

SIRT1 plays an important protective role in diseases associated with vascular dysfunction [46]. In insulin resistance, atherosclerosis, and related coronary artery disease, SIRT1 is an important protective factor that inhibits the abnormal proliferation of vascular smooth muscle in atherosclerosis [47, 48]. Resveratrol is a well-known agonist of SIRT1. For example, resveratrol has been shown to protect mice from diet-induced obesity and insulin resistance, cause improved mitochondrial function, and prevent metabolic diseases by activating SIRT1 [49]. It also induces VSMC differentiation via SIRT1-mediated Akt activation, thereby preventing endothelial regeneration [50]. We investigated whether SIRT1 is involved in the resveratrol-induced inhibition of VSMC proliferation and migration caused by insulin. EX527, a specific inhibitor of SIRT1, abolished the role of resveratrol in inhibiting insulin-induced proliferation and migration and upregulating phosphorylated PI3K and Akt in VSMCs. The findings indicated that resveratrol suppresses insulin-induced VSMC proliferation and migration via SIRT1. SIRT1 exerts many functions in VSMCs, including protecting against DNA damage, inhibiting atherosclerosis [51], inducing cell differentiation [50], and protecting against cell senescence and vascular aging [52]. It seems to play a vital role in the abnormal regulation of VSMCs and may be a potential target in the treatment of vasculopathy induced by various factors, including hyperinsulinemia. Besides SIRT1, resveratrol via SIRT3 also exerts its physiological functions, which could have influence on the present results [53].

Several potential limitations should be considered in interpreting our results. First, our present study was only performed *in vitro*. *In vivo* study would be required to show the inhibitory effect of resveratrol in the proliferation and migration of VSMCs induced by hyperinsulinemia in future. Second, exploration for the underlying mechanism was not much in-depth. For example, how SIRT1 regulates the PI3K/Akt pathway still remains unclear. Third, we only focused on the insulin-induced activation of downstream signaling molecules. However, we did not investigate whether the expression of insulin receptor was changed, which could have triggered the downstream signaling pathway by specifically binding to its ligand insulin. Studies have shown that insulin-mediated VSMC proliferation can be suppressed by inhibition of insulin receptor expression and function in arterial VSMCs [29, 54]. Thus, whether resveratrol can regulate the expression of insulin receptor in VSMCs would require further investigation in future.

## 5. Conclusions

In summary, the current study indicated that resveratrol-activated SIRT1 inhibits insulin-stimulated VSMC proliferation by alleviating the PI3K/Akt signaling axis. Clarification of the precise mechanism by which resveratrol inhibits the proliferation and migration of VSMCs may provide scientific evidence for preventing and treating hypertension and other cardiovascular complications of diabetes in future.

## Data Availability

The data used to support the findings of this study are available from the corresponding author upon request.
